# Exploiting Gene Expression Profiles for the Automated Prediction of Connectivity between Brain Regions

**DOI:** 10.3390/ijms20082035

**Published:** 2019-04-25

**Authors:** Ilaria Roberti, Marta Lovino, Santa Di Cataldo, Elisa Ficarra, Gianvito Urgese

**Affiliations:** Politecnico di Torino (DAUIN), Department of Control and Computer Engineering, Corso Duca Degli Abruzzi 24, 10129 Torino, Italy; ilaria.roberti@studenti.polito.it (I.R.); marta.lovino@polito.it (M.L.); santa.dicataldo@polito.it (S.D.C.); elisa.ficarra@polito.it (E.F.)

**Keywords:** brain connectivity, gene expression, machine learning, Allen Mouse Brain Atlas, classification, prediction

## Abstract

The brain comprises a complex system of neurons interconnected by an intricate network of anatomical links. While recent studies demonstrated the correlation between anatomical connectivity patterns and gene expression of neurons, using transcriptomic information to automatically predict such patterns is still an open challenge. In this work, we present a completely data-driven approach relying on machine learning (i.e., neural networks) to learn the anatomical connection directly from a training set of gene expression data. To do so, we combined gene expression and connectivity data from the Allen Mouse Brain Atlas to generate thousands of gene expression profile pairs from different brain regions. To each pair, we assigned a label describing the physical connection between the corresponding brain regions. Then, we exploited these data to train neural networks, designed to predict brain area connectivity. We assessed our solution on two prediction problems (with three and two connectivity class categories) involving cortical and cerebellum regions. As demonstrated by our results, we distinguish between connected and unconnected regions with 85% prediction accuracy and good balance of precision and recall. In our future work we may extend the analysis to more complex brain structures and consider RNA-Seq data as additional input to our model.

## 1. Introduction

The brain is a complex organ made up of more than 100 billion neurons grouped into many functional regions, that communicate with each other by means of electro-chemical signals. When referring to the brain, physical connectivity refers to the pattern of anatomical links constituted by the neurons’ axons and connected to the dendrites of post-synaptic neurons [1]. The physical connections that link numerous groups of neurons constitute a network that at a larger scale constitutes the so-called anatomical brain connectivity.

It is shown in the literature that functional properties of neurons and neuronal systems depend on neural connectivity patterns [2,3]. This has long attracted the attention of neuro-anatomists, who dedicated their studies to the new field of science dealing with the assembly, mapping and analysis of the connectome [4].

The anatomical connectivity in the brain is constituted by fibers that propagate from the neuronal bodies. These, in turn, contain the nucleus and all the nuclear components that contribute with their activity to the cellular differentiation and morphogenesis. Accordingly, the main factors influencing connectivity patterns have to be searched at the cellular scale, meaning that cellular activity influences physical brain connectivity patterns at the anatomical level. Hence, the analysis of cellular activity in the form of neuronal gene expression profiles may represent an effective way of understanding the physical connectome more in depth [5,6].

Gene expression is the process by which information from a gene is used in the synthesis of a functional gene product such as a protein. Gene regulation gives the cell control over structure and function, and is the basis for cellular differentiation, morphogenesis and adaptability of any organism.

Gene expression profiling is the measurement of the activity (i.e., expression) of genes. Sequence-based techniques such as RNA-Seq provide information on the sequences of genes, from which their expression level can be derived. Nonetheless, they extract information through a disruptive process of the tissue under investigation, providing gene expression levels averaged over the whole cellular population without any spatial information. On the other hand, Single-cell RNA-seq (scRNA-seq) [7], relying on separation of single cells from tissue by enzymatic or mechanical dissociation, provide cell-specific information but even in this case with lack of information on spatial location and the micro-environment [8]. Instead, using in-situ techniques it is possible to detect the spatial distribution of gene expression levels in the tissue. Fluorescence in situ hybridization (FISH or ISH) uses RNA or DNA complementary hybridization probes labeled to fluorescent molecules. Once the probes have hybridized the target in the fixed tissue, the transcript can be localized and quantified through fluorescence microscopy images. Thanks to this process, FISH allows to maintain both spatial and morphological information. On top of that, it generally generates better-quality images than other in situ techniques [7], which makes it the ideal source of information for connectome studies.

Due to the crucial role played by anatomical brain connectivity, scientists generated and made available a number of brain atlases, modelling the axonal connections between different brain regions [9,10]. Upon these connectivity models, the scientific community conducted several studies, aimed at either detecting the existence of anatomical neural connections or spatial correlations between intrinsic properties of the brain tissue. Studies on the mouse brain based on visualization and clustering showed that gene expression and connectivity information have significant levels of spatial auto-correlation, which needs to be accounted for through integrative analysis [11]. Based on the results of these studies, brain regions with similar expression profiles tend to have similar connectivity profiles. Likewise, brain regions which are anatomically connected to each other have gene expression patterns that are particularly similar [11]. Some studies have also identified a set of genes that are responsible for the relationship between cellular activity and connectivity, as they are directly involved in neuronal development and axon guidance [12]. With more in-depth investigations of the specific relationship between gene expression and connectivity in the mouse brain, it was shown that gene expression is predictive of the connectivity pattern when the connectivity signals are in a discrete form. In addition, most of the predictive power resides in the expression data from a relatively small number of genes, suggesting that very few genes are responsible for generating brain connectivity in each specific brain structure [13].

All these findings stem from the analysis of data from the mouse brain. Nonetheless, a large number of genes in the mouse brain find a direct correspondence in the human brain, and regionally enriched genes were demonstrated to be conserved when shifting from one species to another [14]. Based on this evidence, the combination of human and mouse single-cell trascriptomic profiles, through the application of feature selection and linear modeling, was used to provide better insights into human brain connectivity. Then, the combined data were used to demonstrate that gene expression is a better indicator of cellular localization than the location of cell nuclei, especially for cells with large and irregularly shaped cell bodies such as the neurons [15].

Upon these considerations, gene expression data can be effectively used to automatically predict brain connectivity. While most of the works in the literature focus on either analysing the most relevant genetic signatures of neuronal connectivity [16,17], or on investigating the direct relationships between gene expression and brain functionality [18,19,20], lesser attention is devoted to predicting anatomical connectivity at a cellular resolution, directly using the trascriptomic profile as the input baseline. The most representative works in this regard use model-based techniques (e.g., sparse linear models [13,21]), obtaining good prediction accuracy level (between 80% and 90%) at the well-known cost of difficult parametrization and non-obvious selection of the variables.

In this paper, we push forward the path of predicting the degree of anatomical connection of brain areas, by performing an integrative analysis of gene expression profiles and connectivity data. To do so, we interpret the prediction as a classification problem, where the input feature vectors describing gene expression profiles of brain region pairs are automatically grouped into multiple classes based on their level of physical connectivity. To solve this classification problem, we exploit neural networks, which, compared to traditional model-based techniques, have the advantage of being non-parametric, and do not require a priori definition of the mathematical relationships among variables. As such, our tool is developed on a case-study of mouse brain data, but it can be ideally applied to any other application of interest.

Our method implements all the stages that are essential to solve the connectivity classification problem in a fully automated way, including data collection, storage and pre-processing, as well as the in-depth analysis of the prediction outcome, aiming at the investigation of anatomical connections between the brain macro-regions. Based on the nature and complexity of the analysis to perform, we chose to implement a Multilayer Perceptron (MLP), a class of feed-forward artificial neural networks that is often used both for data classification and regression [22]. This network is fed with feature vectors representing the gene expression profiles of two different brain regions. Each element of the feature vector corresponds to a gene, and more specifically to the expression level of that gene in a low-dimensional spot (i.e., a voxel) of a region. To obtain classification labels for the feature vectors, the available spatial connectivity data are aggregated, obtaining a unique value representing the connection between two regions. Then, we investigated the outcome of our network on two different tasks, respectively a multi-class classification task (with three classes corresponding to unconnected, weakly connected and strongly connected areas, respectively) and a simpler binary classification task (unconnected and connected). The analysis was performed on a very large dataset from the cortex and the cerebellum (58 regions in total). These specific regions were selected because the corresponding annotated datasets ensure a very broad representation of connectivity degrees.

## 2. Results and Discussion

In this section we will focus on the results of our methodology. More specifically, we will assess our proposed solution in verifying whether gene expression profiles contain enough information to predict the intensity of anatomical connections between brain regions. On top of that, we will provide a quantitative evaluation of the performance of a classification system leveraging gene expression profiles as input and connectivity as the classification label.

### 2.1. Analysis Pipeline

The proposed methodology consists of an MLP classifier, where the input is a vector (so-called Source-Target vector), representing the gene expression levels of two regions of the mouse brain, respectively called Source and Target. The output of the classifier is a unique categorical label, representing the overall connectivity degree of all the voxels corresponding to the input Source-Target pair.

To generate the Source-Target vectors and corresponding connectivity labels, in this study, we used gene expression and connectivity values available from the Allen Mouse Brain Atlas (AMBA) [23] and the Mouse Brain Connectivity Atlas (MBCA) [9] resources, combined with the connections’ intensities reported by the Brain Architecture Management System (BAMS) database [10].

**Figure 1:**
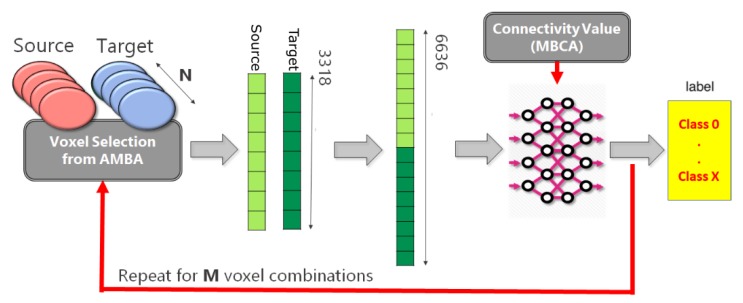
Scheme of the analysis pipeline. For each Source-Target pair (*N*, in total), we randomly select *M* voxel combinations. Per each combination, we generate two 3318 gene expression vectors (Source and Target, respectively) with information taken from Allen Mouse Brain Atlas (AMBA). The concatenation of the two vectors represents the Source-Target vector that is given as input to our Multilayer Perceptron (MLP) model. A categorical label describing the Source-Target connection degree is obtained by setting empirical thresholds on the connectivity values provided by Mouse Brain Connectivity Atlas (MBCA).

By doing so, a very large dataset was generated, choosing as representative brain areas the cortex and the cerebellum. These areas include very important and independent functional regions. Hence, from the analysis of such areas we expect to find (i) dense connectivity between internal sub-regions of the same area and (ii) low connectivity degree between the two areas as a whole. The overall procedure is represented in Figure 1. We considered 58 different regions (eight from cerebellum and 50 from cortex) and randomly selected 21 voxels for each possible Source-Target combination. By doing so, we obtained a total number of 54,495 Source-Target vectors (*M* value in Figure 1), with corresponding connectivity labels. Each of two parts of a Source-Target vector is the expression level of a set of genes within a particular voxel of the mouse brain, where the first and second half of the vector contain values belonging to the Source or to the Target regions, respectively. More details about the databases, as well as on the specific methodology applied to generate Source-Target vectors and labels will be provided in Section 3 and Section 4, respectively.

### 2.2. Classification Performance

The classification consists of a multi-class and a binary classification task, respectively, where the Source-Target vectors are grouped into a corresponding number of categories representing their connectivity degree, leveraging the MLP architectures described in Section 4.3.

#### 2.2.1. Multi-Class Classification Task

To generate a dataset for the multi-class task, all the available Source-Target vectors were divided into three categories based on empirical thresholds on the connectivity values provided by the MBCA database:

1. **Class label “0” (unconnected)**: 5000 Source-Target vectors with connectivity equal to 0
2. **Class label “1” (weekly connected)**: 5000 Source-Target vectors with connectivity values in the range [0.006, 0.1)
3. **Class label “2” (strongly connected)**: 4583 Source-Target vectors with connectivity > 0.1

Therefore, the whole dataset was composed of 14,583 × 6636 vectors with their corresponding class labels. This dataset was randomly split into three disjoint subsets, that were respectively used for training, validation and testing purposes. The three sets contained 10,499 vectors, 1167 vectors and 2917 vectors, respectively.

The MLP architecture implemented to solve the multi-class classification problem will be described in detail in Section 4.3. The training phase consisted of 200 epochs in total, during which the dataset was propagated in batches of size 6. At the end of each propagation, the error between predicted values and desired outputs was quantified in terms of the categorical cross-entropy loss function. Working towards the minimization of the error, Nesterov-accelerated Adaptive Moment Estimation (Nadam [24]) optimizer updated parameters with a learning rate of 0.002 for each training example. To ensure the full reproducibility of the experiment, the full parameter set of the training procedure is summarized in Table 1.

MLP performance was computed in terms of classification errors (i.e., in terms of the fraction of input instances that were correctly assigned to their specific class category). After 200 learning epochs, MLP training accuracy reached an accuracy value on the training set of 0.914, ensuring the convergence of the model. Nonetheless, the classification accuracy of the trained MLP decreased to 0.764 when computed on the test dataset containing completely unseen data, suggesting an over-fitting problem.

To have a more in-depth view of the classification outcome, in Table 2 we show a confusion matrix, with rows and columns representing respectively items in the real and in the predicted class. Hence, the main diagonal of the matrix reports the percentage of instances correctly classified, separately for the three different class categories, while the other elements of the matrix show the misclassified items and their respective distributions.

As it can be gathered from the confusion matrix, the classifier had heterogeneous classification outcome, with best classification accuracy (81%) for the instances with weak connection levels, and lowest accuracy (67%) for the ones with strong connection. Unconnected instances were detected with good level of accuracy (75%). In general, very few misclassifications happened involving two class categories at the extremes: only 2% of the unconnected instances and of the strongly samples were wrongly assigned to the strongly connected class and to the unconnected class, respectively. The most frequent misclassifications (31%) consisted of strongly connected samples classified in the weakly connected class. This is probably due to a slight overfitting of the MLP towards this class, suggesting that the training data were not representative enough for a three-class categorization.

In Table 3, we report the whole set of quality metrics (i.e., recall, precision, F1 score and accuracy [25]) obtained for each class, which confirm the analysis provided above.

If we observe the overall outcome of the classification, we can make the following consideration: while the MLP classifier provides only partial discrimination of the connectivity degree, it has an acceptable accuracy in differentiating between zones with connection (i.e., weakly or strongly connected class) and zones without connection (i.e., unconnected class).

#### 2.2.2. Binary Classification Task

In light of the results obtained in the multi-class predictions, to boost the classifier capabilities in discriminating between connected and unconnected areas, we designed a binary MLP. To perform the binary classification task, this time we divided the available dataset into two sub-sets, as follows:

1. **Class label “0” (unconnected)**: 20,000 Source-Target vectors with connectivity values equal to 0. This sub-set is composed of gene expression vectors obtained selecting only unconnected Source-Target region pairs.
2. **Class label “1” (connected)**: 17,136 Source-Target vectors with connectivity values > 0.006.

Therefore, the overall dataset contained of 37,136 × 6636 vectors, with their corresponding binary labels. Again, the whole dataset was divided into training, validation, and test set, containing respectively 26,737 vectors, 2516 vectors and 7428 vectors.

The training phase consisted of 100 epochs, during which the dataset was propagated in batches of size 32. Again, at the end of each propagation, the error between predicted values and desired outputs was calculated by the binary cross entropy loss function. The learning procedure leveraged the Nadam optimizer, updating parameters with a learning rate of 0.002 for each training example. The overall set of the training parameters are shown in Table 4.

**Figure 2:**
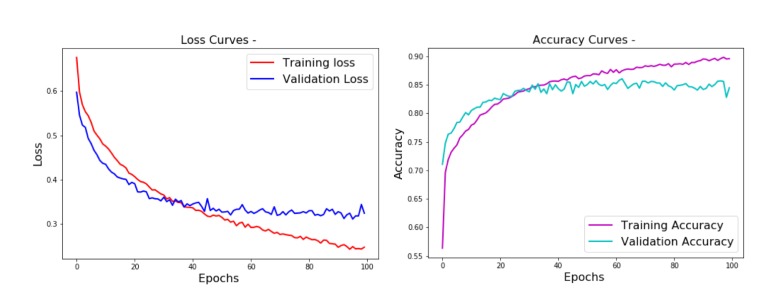
Training performance curves (loss on the **left**, accuracy on the **right**) of the binary classifier.

As shown in the training curves of Figure 2, after the 100 epochs of training, the MLP reached 0.89 training accuracy with 0.247 loss. The validation accuracy on the other hand turned out to be not much lower than the training accuracy (around 0.85), suggesting a correct convergence without overfitting.

To assess the performance of the trained MLP on both the classes, besides accuracy we quantified precision, recall and F1 score on the test set (see values reported in Table 5).

As it can be gathered from the table, the overall classification outcome was positive (85% accuracy), with reasonable balance between precision and recall in both the class categories. The unconnected class has better precision value (94%) and vice-versa the connected class has higher recall (95%), but both classes have similar high values of F1 scores (84% and 85%, respectively).

**Figure 3:**
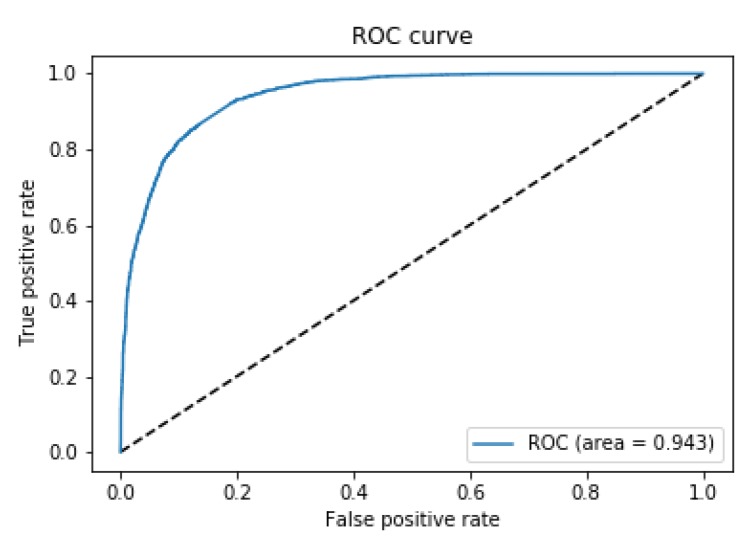
Receiver operating characteristic (ROC) curve on the test set for binary classification.

The good performance of the classification system is also confirmed by the shape of the ROC curve (in Figure 3), with area under the curve equal to 0.943.

This last experiment demonstrates that it is possible to distinguish between connected and unconnected regions in a reliable way. The fine discrimination between different intensities of physical connections is also possible, but with more uncertainty most probably due to technological noise of the training data.

## 3. Materials

In the following, we describe in more detail the datasets from which the Source-Target vectors given as input to our MLP model and corresponding connectivity labels shown in Figure 1 were obtained.

### Allen Mouse Brain Atlas

The Allen Mouse Brain Atlas (AMBA) represents an integration between transcriptomic and neuroanatomic mouse brain data. It is a complete high-resolution atlas of gene expression throughout the adult mouse brain composed of different sections and tools that enable an easy data navigation and analysis. Gene expression patterns are available as images obtained by in-situ hybridization (ISH) technique [26] applied on full brains of 56-day old C57BL/6J male mice. For each gene, expression levels are provided as grid data, in the form of a 3D matrix representing the three-dimensional structure of the mouse brain. Each element of the matrix is a voxel at 200 μm resolution, storing a gene expression level. In our study, this information is used as input to our classification model.

**Figure 4:**
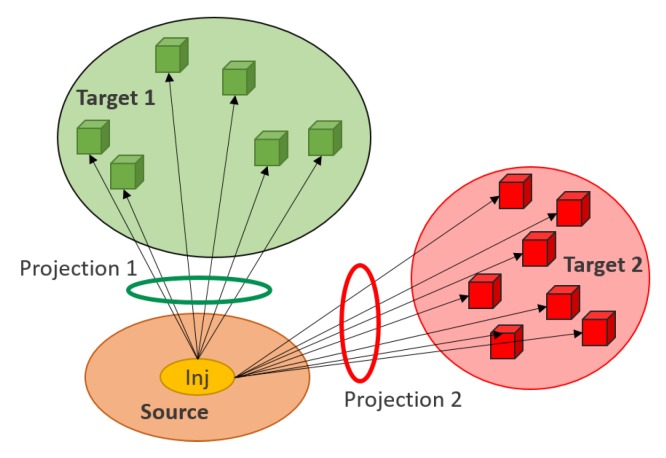
Schematic diagram of Source-Targets projections. A Source is a brain region where the viral tracer is injected (inj, in the figure). As a result of the injection, multiple axonal projections are produced in so-called Target regions.

The Allen Mouse Brain Connectivity Atlas (MBCA) consists of connectivity values in the form of axonal projections labeled by rAAV, that is a viral tracer injected in a specific site and then detected through two-photon tomography. When the viral tracer is injected in a brain region, referred to as Source region, it produces axonal projections in several Target regions (see Figure 4 for a schematic representation). These projection data are provided for more than 200 mouse brain regions, in coronal section. In the Allen MBCA database, more than one injection site can be found for a single brain region. In Section 4.1, we will describe in detail how multiple injection sites were handled in our proposed methodology.

Similar to gene expression, connectivity information is available for each injection site in the form of grid data. Each element of the corresponding 3D matrix is a voxel (in this case, provided at 100 μm resolution) whose value represents the connectivity degree in that specific 3D position. In our study, the connectivity data is used to obtain a classification label for each couple of gene expression profiles coming from two different brain regions.

Corresponding gene expression and connectivity data (respectively from AMBA and MBCA) of each brain region can be coupled at different spatial resolutions, using structural annotation files. The cerebral regions, grouped into hierarchical layers, consist of several voxels of gene expression and connectivity values both referred to a reference space created by the Allen team for mouse brain modeling.

As additional source of information for our study, we used neural circuitry data provided by BAMS to select the most significant brain areas for our investigation. This database contains about 45,000 connection reports between different gray matter regions of the rat, in the form of interactive matrices showing the strength of connection of each brain region pair. Several studies demonstrate that mouse and rat brains share the same anatomical features, only at a different scale [11,27]. Hence, the connection reports can be used to select the most promising brain areas even in the mouse.

## 4. Methods

Besides the MLP prediction model, we implemented a complete automated pipeline to handle dataset collection, as well as the organization and processing of the gene expression and connectivity data into Source-Target vectors with corresponding class labels, to be given as input to the MLP. The main steps of this pipeline, implemented exploiting the Knime framework [28] and the SeqAn library [29], are the following:

1. download of grid data from the available data sources;
2. processing of the raw grid data to integrate the gene expression and the connectivity information;
3. generation of a full and coherent dataset of Source-Target gene expression vectors and corresponding connectivity labels, ready to be cropped into training, validation and test sets for the MLP.

### 4.1. Download of Grid Data

The Allen Brain Atlas provides grid-data at different resolutions, consisting in 3D summaries of both the gene expression and connectivity data, re-sampled to a Common Coordinate Space of the 3D reference brain model [30]. To enable spatially coherent processing of these two sets of data, the database provides a structural grid data annotation system at each resolution scale. This annotation allows to link mouse brain voxels to anatomical structures in the Common Coordinate Space.

Grid data is downloadable through an API service by queries. The queries were implemented through a web application (the RMA BUILDER), that is freely accessible on the Allen Brain Atlas’s API section.

#### 4.1.1. Gene Expression

As mentioned in Section 3, the Allen Institute Mouse Gene expression data consist of whole-brain in-situ hybridization data obtained from brains of 56-day old C57BL/6J male mice [31]. The grid-data of the detected expression levels are provided for coronal and sagittal sections. Even though the sagittal section counts more than 20,000 genes, connectivity data are available only for the coronal section. Thus, for our study we focused on the 3318 gene expression grid-data corresponding to this specific section.

**Figure 5:**
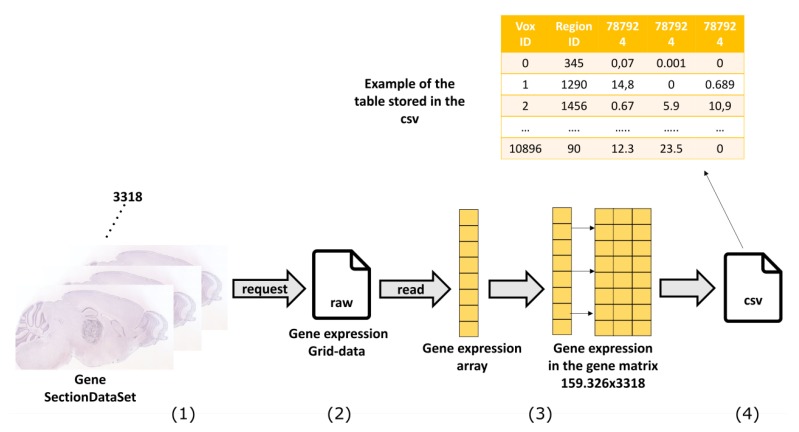
Elaboration of gene expression data: main phases. (**1**) Retrieve a SectionDataset for each of the 3318 genes; (**2**) download grid expression data in the form of an energy.raw file; (**3**) reconstruct a 3318 × 159,326 matrix of gene expression levels, with rows corresponding to genes and columns to 3D voxels; (**4**) store data into a .csv file.

**Figure 6:**
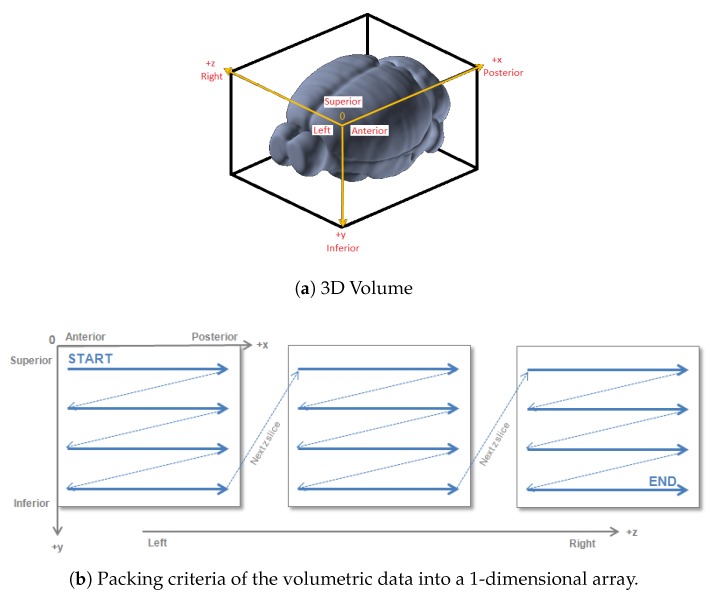
(**a**) 3D Volume of the mouse brain; (**b**) The common reference space is in PIR orientation where *x* axis = Anterior-to-Posterior, *y* axis = Superior-to-Inferior and *z* axis = Left-to-Right.

The main phases of the elaboration of gene expression data are represented in Figure 5. The expression profile of each gene throughout the mouse’s brain is associated to a SectionDataSet, a specific data object of the Allen Brain Atlas framework where all the experiment’s information is stored. We first build a query to retrieve the SectionDataSet unique identifiers (IDs) for the gene expression experiments in the form of an XML document. Then, to retrieve the corresponding gene-expression grid-data, we build a query with the RMA BUILDER and obtain in return an energy.raw file for each of the 3318 gene expression experiments. This file contains a vector of 159,326 elements corresponding to the 3D voxels of the mouse brain model (67 × 41 × 58 voxels at 200 μm resolution) that can be reconstructed leveraging the reference information provided by the database, as shown in Figure 6 [32]. At the end of the download procedure from the Allen Brain website [33], we obtain a 3318 × 159,326 matrix of gene expression levels, with rows corresponding to genes and columns to 3D voxels. This matrix is stored into a single .csv file, as represented in Figure 5.

#### 4.1.2. Allen Connectivity

As outlined in Section 3, The Mouse Brain Connectivity Atlas provides connectivity information in the form of axonal projections labeled by rAAV viral tracer and detected through two-photon tomography for more than 200 mouse brain regions in coronal section. Injection sites refer to the spots where the viral tracer is injected. The region where a certain injection site is placed and the region where the injection produced axonal projections are referred to as Source and Target regions, respectively. Injections involving a single region are called primary. Nonetheless, because of the small size of the mouse brain, a single injection can involve more than one region. These are called secondary injections.

In this work, we focused only on the primary injection sites, and considered connectivity data at 100 μm resolution, that is the closest to the 200 μm gene expression resolution among all the available ones (10, 25, 50, 100 μm, respectively).

**Figure 7:**
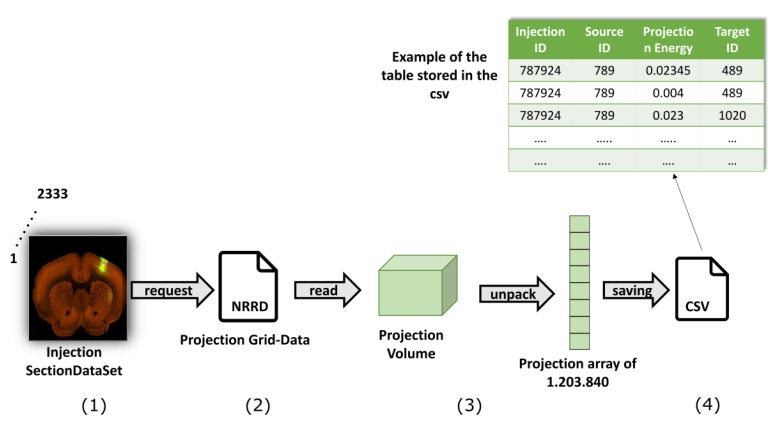
Elaboration of connectivity data: main phases. (**1**) Retrieve a SectionDataset for each of the 2333 primary injections; (**2**) projection grid-data in the form of an .Nrrd file; (**3**) reconstruct a projection volume, unpacked into a vector of 1,203,840 elements; (**4**) store data into a .csv file.

The main phases of the elaboration of connectivity data are represented in Figure 7. Again, each primary injection site corresponds to a SectionDataSet. Hence, we first designed a query to retrieve the SectionDataSet IDs of injection experiments through the API service, which returns an XML document with 2333 primary injection IDS. Such IDs are exploited to build a query with the RMA BUILDER and retrieve the connectivity grid data in return. By doing so, we obtain 2333 .Nrrd files, each representing the axonal projections of a specific primary injection site. This provides a correspondence between the 2333 primary injections and their corresponding target regions. For connectivity data, the 3-D volumetric grid-level information at 100 μm are provided in the form of a 13 × 80 × 114 numerical array, as represented in Figure 6a. Maintaining the spatial reference provided by the Allen Brain Atlas, each 3D matrix was unpacked into a vector of 1,203,840 elements. This way, we obtained 2333 vectors in total, that were stored into a single .csv file along with the source region indication (see last phase of Figure 7).

#### 4.1.3. Structural Annotation File

An annotation volume is a 3D raster image that partitions the reference space into a number of structures, whose number of voxels depends on the size of the structure as well as on the specific resolution of the model. Each voxel is assigned to a specific brain structure by means of a region ID [34]. Brain structures in the Allen reference spaces are arranged in trees, with leaf nodes representing very fine anatomical partitioning and nodes closer to the root corresponding to gross partitioning. The annotation file reports region IDs together with the details of the finest anatomical partitioning. Hence, gene expression and connectivity data can be mapped to several common reference spaces. To link each data voxel to the corresponding membership brain region, the Allen Brain Atlas provides a structural annotation file at different resolutions, where the *i*-th annotation element allows to map the *i*-th voxel in the data array to its brain structure.

Same as for the gene expression data, the annotation is provided at 200 μm resolution, in the form of a vector of 159,326 elements. Likewise, the connectivity annotation (CA) is provided at 100 μm resolution, reshaped in the form of a vector of 1,203,840 elements. Since, differently from the other data, the primary injection structure annotation is not provided at the finest annotation level, we implemented a procedure to trace both the annotations back to the same resolution level, in the annotation tree. To do so, we exploited a list of dictionaries provided by the Allen Brain Atlas, documenting brain structures and their hierarchical relationships in the form of a structure graph.

#### 4.1.4. Brain Architecture Management System (BAMS)

**Figure 8:**
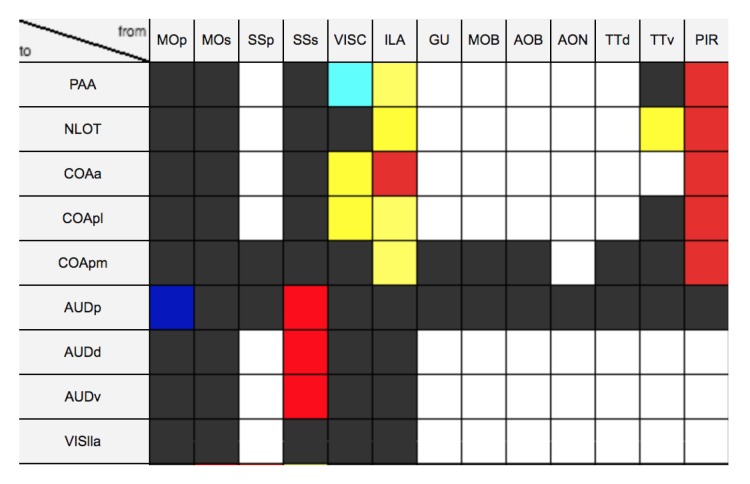
Interactive matrix from Brain Architecture Management System (BAMS). Each element of the matrix represents the connection between two regions, reported in rows and columns. Different colours encode different connection intensities, with white corresponding to unknown connections.

As mentioned in Section 3, additional neural circuitry data collected from BAMS were used as reference to decide which brain structures are most significant for our analysis. To date, BAMS includes about 45,000 connection reports between different gray matter regions, leveraging information on connections that were demonstrated by previous studies. The reports can be freely downloaded from the site of BAMS [35] in the form of an interactive matrix (see Figure 8), where each element (i,j) defines the existence and the intensity (encoded by a value in a <1–9> range) of the connection between two specific brain regions *i* and *j*, identified by the same universal acronyms used by the Allen Brain Atlas. Unknown connections are assigned a 0 value.

### 4.2. Generation Source-Target Vectors and Corresponding Connectivity Labels

The last step of the dataset generation consists in assigning a unique connectivity label to the Source-Target gene expression vectors, as follows.

All the connectivity values reported for a specific injection ID (i.e., experiment) and a specific Source-Target combination are first aggregated based on their median value. This solution is preferred to others (e.g., mean value), because the median value is inherently robust to the presence of outliers and noise. Nonetheless, as in the nature of this technology, a specific region may be a site of injection of multiple experiments. Hence, for each of these experiments, the connectivity of the axonal projections produced in the corresponding target regions will be stored in a specific SectionDataSet. Then, in case a specific source has targeted the same region in different experiments, that specific combination of Source-Target regions will correspond to more than one median value. To tackle this issue, we implemented a second level of aggregation, and obtained the final connectivity value as the maximum of all the multiple median values. This choice stems from the empirical observation that the connectivity network detected in each experiment (and hence, the corresponding connectivity value) is highly dependent on the specific position of the injection. Hence, using the maximum as the most representative value has a two-fold advantage: (i) it filters out small connectivity values possibly due to peripheral injection sites and (ii) allows to select the experiments with the best spatial conditions as the most representative of a specific source-target combination.

Based on the empirical connectivity thresholds defined in Section 2, this connectivity value is transformed into a categorical label representing the strength of the connection: either (0,1,2) for multi-class classification, or (0,1) for binary classification.

**Figure 9:**
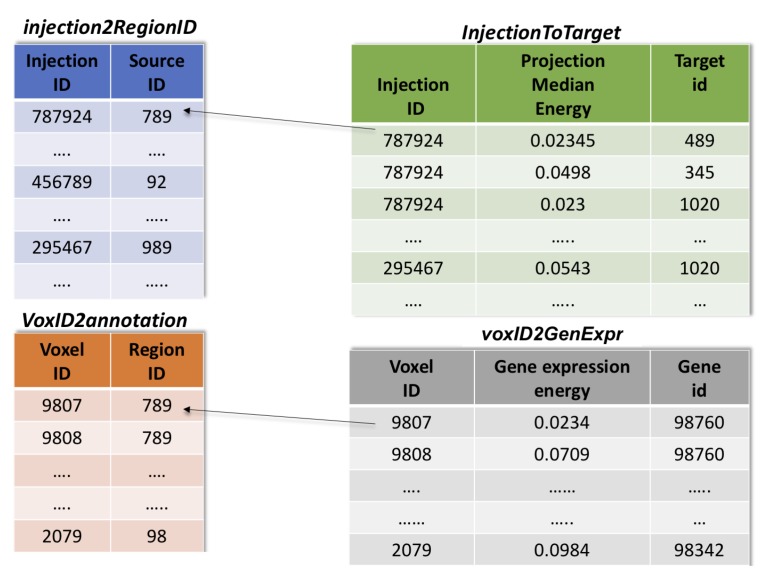
SQLite database tables generated to store all the gene expression and connectivity data.

To allow further processing and easy access of the data, in our solution the full and coherent dataset of Source-Target gene expression vectors and the corresponding connectivity labels were stored into four tables of an SQLite database shown in Figure 9:

1. Table voxID2Annotation carries the spatial information, and contains the voxel ID and corresponding brain structure annotation.
2. Table voxID2GenExpr was obtained by filtering out the voxels with gene expression level value equal to 0. It is made of columns reporting gene expression value, voxel ID and gene ID, respectively.
3. Table injection2regionID was obtained by grouping all the voxels by Source and injection ID. Hence, it reports the Source region ID for each injection.
4. Table injection2target was obtained by grouping the connection values of each voxels by the Target ID. More specifically, all the voxels belonging to the same Target region were aggregated by the median of the values associated to each of these voxels. Then, the final table is composed of three columns: injection ID, median of the values obtained for a specific Target ID and its annotation ID, respectively.

This database solution allows the quick generation of custom datasets to be given as input to prediction models, avoiding the need to re-process the raw-data.

Leveraging such database, a custom dataset can be built as follows. First, *N* Source-Target regions are selected, based on the specific analysis to perform. Gene expression and connectivity data of the selected pairs undergo the following pipeline, as represented in Figure 1:

1. for each Source-Target pair, *M* voxels belonging to the source region and *M* voxels to the target regions are selected on the expression gene annotation.
2. for each selected voxel, a vector composed of 3318 elements is generated, where each element corresponds to the expression level of a specific gene. Hence, *M* vectors representing the gene expression profile of the Source and *M* vectors representing the gene expression profile of the Target are obtained.
3. A dataset is created by selecting *P* combinations among all possible Source-Target voxel combinations. More specifically, the gene expression vector corresponding to the Source voxel is concatenated with the gene expression vector corresponding to the Target voxel. Hence, the obtained dataset will be made of *P* vectors.
4. In the end, a unique categorical label representing the Source-Target connectivity is assigned to each combination.

These steps are repeated for all *N* number of Source-Target regions.

The obtained dataset undergoes a normalization process, by scaling input vectors in a (0,1) range. Then, they can be divided into training, validation and test sets, to be fed into the predictive model.

### 4.3. MLP Predictive Model

As a predictive model, we designed a Multilayer Perceptron. In the following, we describe in detail the MLP architectures and corresponding design parameters that provided the best performance values for the multi-class and binary classification tasks discussed in Section 2.

**Figure 10:**
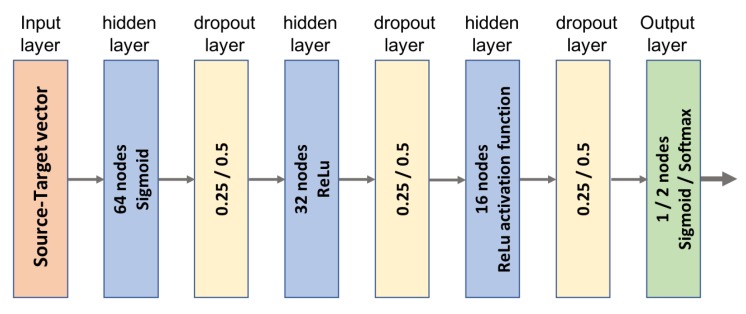
MLP architecture for classification tasks. For each layer, we report number of nodes, activation function and dropout value. When values are different for binary and multi-class tasks, we report them both, separated by a slash symbol.

This MLP architecture, represented in Figure 10, is composed of a hidden layer with 64 nodes and two hidden layers with 32 nodes each. The first hidden layer applies a ‘sigmoid’ activation function on the entries. In the following hidden layers, nodes apply the ‘ReLu’ (rectified linear unit) activation function on their inputs. Three Dropout layers are placed after the hidden layers to avoid the overfitting phenomenon, occurring when the MLP specializes too much on the training set losing its ability to generalize on the validation set. When the error on the validation set starts to increase, indicating possible overfitting, the dropout layers “drop out” random neurons, temporally removing their contribution to downstream the activation of neurons. This has been widely demonstrated to improve the generalization capabilities of the network [36]. Notably, two options are given for the activation function of the output layer: softmax and sigmoid, respectively for multi-class and binary classifications.

## 5. Conclusions and Future Work

As demonstrated by our results, our gene expression data-driven approach allows to distinguish between connected and unconnected brain areas at a cellular resolution scale, with no need for extensive parametrization or a priori knowledge of the process.

This opens the way to more in depth investigations on the genetic footprint of brain connectome and brain functionality. The possible directions of this study are mainly two. The first is aimed at extending the available knowledge on brain connectivity. Indeed, structural information on neural circuitry (e.g., BAMS) is to this date characterised by a large number of unknown connections and missing data. In the long term, the second direction of our work will be the investigation of the transferability of the connectivity prediction model from mouse to other mammalians (especially humans). In this regard, the main research question to be answered is how and to which extent the prediction model trained on the Allen Mouse Brain Atlas can be applied (either as-is or after partial fine-tuning of the network on new training data) to infer the anatomical connectivity of more complex brains, possibly exploiting not only gene expression levels from in-situ hybridization but also RNA-Seq data.

## Figures and Tables

**Table 1:** Training parameters for multi-class classification with the Nadam optimizer.

Epochs	Learning Rate (Lr)	Decay	Beta1	Beta9	Loss Function	Batch Size
200	0.002	0.004	0.9	0.999	categorical cross entropy	6

**Table 2:** Confusion matrix for multi-class classification.

		Predicted Class		
		Unconnected	Weakly Connected	Strongly Connected
	**Unconnected**	75%	23%	2%
**Real Class**	**Weakly Connected**	13%	81%	6%
	**Strongly Connected**	2%	31%	67%

**Table 3:** Quality metrics for multi-class classification.

		Quality Metrics			
		Recall	Precision	F1_Score	Accuracy
	**Unconnected**	75%	86%	81%	
**Class**	**Weakly Connected**	81%	66%	73%	76%
	**Strongly Connected**	67%	83%	74%	

**Table 4:** Training parameters for binary classification with the Nadam optimizer.

Epochs	Learning Rate (Lr)	Decay	Beta1	Beta9	Loss Function	Batch Size
100	0.002	0.004	0.9	0.999	binary cross entropy	32

**Table 5:** Quality metrics for binary classification.

	Quality Metrics			
	Precision	Recall	F1_Score	Accuracy
**Unconnected**	94%	75%	84%	85%
**Connected**	77%	95%	85%	

## Abbreviations

The following abbreviations are used in this manuscript:

ISH	In-Situ Hybridization
AMBA	Allen Mouse Brain Atlas
BAMS	Brain Architecture Management System
MBCA	Mouse Brain Connectivity Atlas
MLP	Multilayer Perceptron
